# African Primary Care Research: Choosing a topic and developing a proposal

**DOI:** 10.4102/phcfm.v6i1.580

**Published:** 2014-02-06

**Authors:** Bob Mash

**Affiliations:** 1 Division of Family Medicine and Primary Care, Stellenbosch University, South Africa

## Abstract

This is the first in a series of articles on primary care research in the African context. The aim of the series is to help build capacity for primary care research amongst the emerging departments of family medicine and primary care on the continent. Many of the departments are developing Masters of Medicine programmes in Family Medicine and their students will all be required to complete research studies as part of their degree. This series is being written with this audience in particular in mind – both the students who must conceptualise and implement a research project as well as their supervisors who must assist them.

This article gives an overview of the African primary care context, followed by a typology of primary care research. The article then goes on to assist the reader with choosing a topic and defining their research question. Finally the article addresses the structure and contents of a research proposal and the ethical issues that should be considered.

## Abstrait

**Recherche africaine en soins de santé primaires: choisir un sujet et développer une proposition**. Voici le premier article d'une série sur la recherche en soins de santé primaire dans le contexte africain. L'objectif de cette série est de renforcer les capacités de recherche en soins de santé primaire parmi les départements émergents de médecine familiale et de soins de santé primaire sur le continent. Beaucoup de départements développent actuellement des programmes de Masters de Médecine en Médecine familiale, et leurs étudiants devront effectuer des recherche dans le cadre de leur diplôme. Cette série est rédigée en ayant ce type de lecteurs particulier à l'esprit: les étudiants qui doivent conceptualiser et mettre en place un projet de recherche, ainsi que leurs superviseurs qui doivent les aider.

Cet article donne un aperçu du contexte africain de soins de santé primaire, suivi d'une typologie de recherche en soins de santé primaires. La suite de l'article aide le lecteur à choisir un sujet et à définir sa question de recherche. L'article s'achève en traitant de la structure et des contenus d'une proposition de recherche et des questions éthiques qui doivent être prises en compte.

## Introduction

This is the first in a series of articles on primary care research in the African context. The aim of the series is to help build capacity for primary care research amongst the emerging departments of family medicine and primary care on the continent. Many of the departments are developing Masters of Medicine programmes in Family Medicine and their students will all be required to complete research studies as part of their degree. This series is being written with this audience in particular in mind – both the students who must conceptualise and implement a research project as well as their supervisors who must assist them. The emergence of a group of doctors, all of whom are required to perform primary care research, is also a major opportunity for the strengthening of primary healthcare.

## The African context

Leading causes of the burden of disease in the African region include HIV, lower respiratory tract infection, diarrhoeal disease, malaria, neonatal infections, birth asphyxia, prematurity, tuberculosis, road traffic accidents and malnutrition.^1^ Some countries, such as South Africa, also have a high burden of disease from violence and non-communicable chronic diseases.^1, 2^ Africa has the lowest life expectancy of all the world's regions.^1^

The African region has the lowest numbers of doctors, nurses and midwives per capita and primary healthcare systems are particularly underdeveloped.^1^ The World Health Assembly has called for a multidisciplinary team approach to primary healthcare that would include community health workers, nurses, midwives, family physicians and allied health professionals.^3^ The World Health Organization has called for a re-commitment to primary healthcare and has emphasised the need for four reforms: universal coverage to improve equity, service delivery reforms to make health systems more people centred, leadership reforms to make health authorities more reliable and public policy reforms to promote and protect the health of communities.^4^ It is clear that the challenges facing primary care providers are immense, both in terms of the burden of disease in the people they care for and the organisational issues in the health systems they work within. The need for primary care research to assist in overcoming these challenges is also clear.

It is, however, difficult to generalise about Africa because of its vast geographic size and its huge variety of cultures, health systems and health needs. The intention of this article is not to suggest a list of research priorities or topics, but to present a conceptual framework for thinking about primary care research in our context. I will also outline the practical process of deciding on a topic and writing your research proposal.

## Primary care research

Primary care research can be characterised as research that is not only about primary healthcare, but also conducted within and even by primary care providers. In the African context, one might also speak of research conducted in the health district as family physicians may also work as generalists in the district hospital.

One of the limitations of evidence-based medicine applied to the primary healthcare context is that much of the evidence is derived from hospital-based settings and populations and is difficult to generalise to primary care. Primary care has many differences from the hospital context, for example the range of conditions seen is different, the probability of severe disease in any encounter is far lower and providers are often managing multiple problems simultaneously. There is therefore a need for research to be conducted in primary care and to produce a more relevant evidence base. When thinking about primary care research, one can categorise it into the following types.^5^

### Basic

Basic research has been defined as studies that ‘help develop the research tools for primary care’.^5^ The International Classification of Primary Care is one such tool that allows for the systematic coding of reasons for encounter, diagnoses and episodes of care.^6^ More modest examples include the development of a 360-degree evaluation tool for assessing the impact of family physicians on the local health system or adaptation of the Primary Care Assessment Tool to local primary care systems.^7^

### Clinical

Clinical research focuses on the burden of disease and the clinical processes involved. This may include the natural history, epidemiology, diagnosis and management of common clinical problems. Studies at a Masters level often focus on improving the quality of care or implementing guidelines for specific conditions or on understanding more about the patient's experience of care or perspective on their health. Large-scale clinical trials and observational studies are not usually feasible at a Masters level unless several students are co-opted as research assistants to contribute parts toward a larger study. Translational research may focus on whether an intervention is feasible in low-resource settings and whether the resultant benefits are worth the cost. Sometimes a systematic review may address a relevant specific clinical question.

### Health services

Health services research focuses on the way primary care is organised and the processes involved. Key attributes of primary care, which cut across multiple clinical conditions, may be looked at, for example:^8^

- Access to primary care services: Availability of services, geographic accessibility, how care is organised in order to make it more accessible, affordability, acceptability of services to patients, actual utilisation and equality of access for different groups.
- Continuity of primary care: How well does the health service care for a patient over time and multiple illness episodes, what is the nature and quality of the relationship with the healthcare providers and how is the incremental information organised and accessed in the medical record?
- Coordination of primary care: How well does the health service coordinate a patient's care within and between levels of care? For example, between community-based and facility-based services, or between primary care and district hospitals, or with secondary care. How is care coordinated within the primary care team or facility?
- Comprehensiveness of care: Is the full range of services available to meet all the patient's health care needs?

### Health systems

Health systems research is more at a policy level and may look at the relationship of health policies to other political, social or economic systems. It may also look at how policy is implemented and the impact on services, equity and health outcomes. For example, many of the human resource challenges for primary care are health systems issues, including the roles and responsibilities of the family physician in that system.

### Educational

Research in this area focuses on the development of educational programmes for family medicine and primary care and continuing professional development. For example, research may look at the clinical skills that should be taught, the learning outcomes required or the use of learning portfolios. Research may also look at recruitment and retention of professionals, as well as career pathways.

## Choosing a topic

Having outlined a broad typology of the different kinds of primary care research that are possible, the postgraduate student must eventually decide on a single topic for their research project. This section discusses some of the key considerations in choosing a topic.

One of the easiest routes for a postgraduate student is to become involved in a larger study that an established researcher has already designed. The advantage for the student is that they do not have to conceptualise the project from scratch, the researcher may already have obtained funding for the work and the work may have a higher impact. The advantage for the researcher is that they can expand the scope of their work through the use of multiple research assistants, build research capacity and produce a more significant piece of work.

Even if such a large-scale project is not available it can be helpful for key stakeholders and established researchers to suggest priorities to the students. At a local level the family physician trainer, facility or district manager and the student may discuss how best to use the opportunity for a research project to take things forward. The university and the department of health may also have documents outlining research priorities and key focus areas.

Often a student will generate potential research questions from reflection on their own challenges and experience in service delivery. Sometimes reading the scientific literature may prompt questions, seeing as most original research articles discuss the need for further research and unanswered questions. At an individual level, the student can judge the value of pursuing any research question by considering the following issues:

- How passionate or interested am I in this topic?
- What is the social value of this topic – how relevant or important is it to health needs in my context? Will answering this question make a difference?
- What is the scientific value of this topic – do I already know the answer to this question? What is the original contribution of this study to new knowledge? Sometimes the contribution to new knowledge is within the African context, even though the question may have been addressed elsewhere. It may be necessary to explore the literature before answering this question. Exploring the literature often leads to a new focus or more original question within the same area of interest.
- How feasible is it for me to answer – can I answer this question given my time, expertise and available resources? Students are often overly optimistic about the type of study design that is feasible. Clinical trials are often suggested, but in reality most Masters students do not have the time, financial resources or expertise to be a principal investigator of such a trial.

It can be helpful to actually rate your answers to the questions above to compare multiple possible research questions. It is usually helpful to discuss your ideas with your supervisor or a more experienced researcher. All comments and suggestions are worth considering seriously although you should not abandon your own viewpoint too easily.

## Writing your research proposal

The following structure is widely accepted as being suitable for a research proposal.^9^

### Project title

Your title should make clear the purpose of the research and sometimes the study design as well. It is likely that it will change as your research proposal evolves. Ensure that the final title is not too lengthy or obscure.

### Cover page

Provide your name, student number, registered course, supervisors, collaborators, affiliations and contact details, that is, address, telephone, fax and email address.

### Summary

You may well find this easier to write at the end, once you are clear about the project. It should summarise the project succinctly and stimulate interest in the topic. The summary is important because this may be the only part that key people look at, for example members of the ethics committee other than the designated reviewers or facility managers who must give permission for the research. The summary should also be structured into, for example, background, aim and objectives, methods and ethical considerations. It should not be longer than one to two pages.

### Introduction

The introduction section to your proposal should convince the reader of the social and scientific value of your study. It would be unethical to conduct research that has no social or scientific value. You must construct a logical argument that utilises your review of the literature to support your argument for the social and scientific value of the research. In some studies it will also be important to present the theoretical basis and conceptual framework for the study. How to search, obtain and appraise the literature for this purpose, as well as how to construct this argument, are discussed in another article in this series. Note that no separate section for a literature review is proposed; instead, the literature is aligned with a specific purpose in the introduction.

### Aim and objectives

You should define the main research question that you intend to answer and any hypothesis that will be tested. Your aim is often the same as your research question, but expressed as a statement rather than a question. You can then break down the broad aim into a number of secondary components or objectives. Be very careful that your choice of words here is an accurate reflection of what you intend to do (i.e. explore, measure, compare, evaluate). Your objectives are really aspects of the research question that the study will answer and should not be a description of the methodological steps. In the conclusion to your thesis you will conclude in terms of your findings for each of these objectives. Table 1 gives some examples of research questions, aims and objectives. If your research question is clearly formulated, the correct study design will be much easier to identify. However, if the research question is unclear, it is almost impossible to choose a correct design.

**TABLE 1:** Examples of research questions, aims and objectives.

Examples	Research question	Aim	Objectives	Study design
Example 1	What do health-promotion officers think and feel about their implementation of a group diabetes education programme?	To explore what health-promotion officers think and feel about their implementation of a group diabetes education programme.	To explore their experience of the training they will receive on how to deliver and facilitate the education programme. To explore their views on the group diabetes education programme: the design of the programme, the resource materials provided, the information on diabetes, the facilitation style, the response of patients and any other issues.	Qualitative focus group interviews.
Example 2	What is the prevalence of substance abuse and its risk factors amongst adolescents attending high schools in the Example area?	To determine the prevalence of substance abuse and its risk factors amongst adolescents attending high schools in the Example area.	To determine the prevalence of tobacco smoking and use of alcohol, methamphetamine, cannabis, heroin, cocaine and any other illicit substances amongst adolescent high school students in the Example area. To evaluate the relationships between tobacco smoking, alcohol, cannabis and other illicit drug abuse. To evaluate the importance in this context of known factors associated with substance abuse. To evaluate any gender differences relevant to substance abuse.	Cross-sectional survey.
Example 3	How to improve the quality of care for congestive cardiac failure at Example Community Health Centre?	To assess and improve the quality of care for congestive cardiac failure at Example Community Health Centre.	To assess the current quality of care for congestive cardiac failure. To plan and implement changes to improve the quality of care. To assess if these changes are associated with a measurable improvement in the quality of care.	Quality improvement cycle.

### Methods

In this section, you need to consider the following aspects:

#### Study design

What type of design is appropriate to achieve your aim and answer your research question? The methods section will vary according to the type of study design and whether it is within an empirical-analytical paradigm (usually involving quantitative techniques such as randomised controlled trials, diagnostic studies, case-control studies, cohort studies or cross-sectional surveys), an interpretive-hermeneutic paradigm (usually qualitative techniques such as in-depth interviews or focus group interviews) or an emancipatory-critical paradigm (participatory action research). Many universities will also accept quality improvement cycles and project evaluations at a Masters level. It should be a clear and well-structured account of what you are going to do, of how you will establish the quality of what you plan to do and of any anticipated problems or limitations. Other articles in this series will discuss in detail the common study designs used by postgraduate family medicine students.

#### Setting

Describe the setting of the research study and study population. These could include, for example, the characteristics of the community involved as well as the facilities, staff and services available in the health system where the study is located.

### Study population and sampling strategy

This refers to the manner in which you select people for the study or assign them to control or intervention groups. The study population should be defined in terms of who they are, where they are and any time periods. Define any inclusion or exclusion criteria. The size of the sample from this study population that you want to include in the study should be defined and justified. In quantitative studies, this requires a full explanation of the assumptions used in the calculation and usually needs the help of a statistician or at least advice from your supervisor. For simple sample-size calculations you can use an online statistical calculator or free software (for example, http://wwwn.cdc.gov/epiinfo/html/downloads.htm). The statistical issues vary depending on whether it is a representative sample, as in a survey, or whether a comparison of groups is involved. In qualitative studies, this relates to issues of saturation, whereby no new themes are being generated from further sampling. Once the sample size is clear then the process of obtaining this sample must be described. For example, will selection be random, systematic (every nth person) or purposeful and based on pre-defined criteria? How, in practical terms, will selection be done – by whom, where and how?

#### Interventions

In the case of an experimental-type design, describe the interventions that you will make on different groups.

#### Data collection

Describe the types of measurements or data collection methods that you will use. The validity of tools, such as questionnaires, needs to be established or you must describe how you will address this. It is usually better to use or adapt tools that have been developed and validated previously. The data collection tools such as interview guides or questionnaires are usually included as an appendix. This section must also describe in practical terms the process of data collection – who will be involved, where this will happen, how they will do it and how language barriers will be addressed. Future articles in this series will focus on the use of questionnaires and qualitative interviews.

#### Data analysis

Describe how you will capture and store your data. Describe how you will check and clean your data prior to analysis. Describe the way in which you will analyse this data. Describe any software that you will use. You may need to consult a statistician for help to determine the appropriate statistical tests to use. Describe the steps in qualitative data analysis. Future articles in this series will focus on quantitative and qualitative data analysis.

### Ethical considerations

Before embarking on any research study it is necessary to consider the ethical challenges. There are many examples in Africa of vulnerable communities being abused by researchers and of research findings being misrepresented. The following seven-point checklist has been developed specifically for health research in developing country settings:^10^

#### Social and scientific value

- Is the research question relevant?
- Is the research that is going to be reported been done previously? Sometimes, even if it has not been done in the researcher's own setting, it can still have scientific value.

#### Scientific validity

- Is the design and methodology of the study sound?
- Has the analysis process been considered?
- Is the sample size justified?

#### Fair selection of study population

- Has the study population been chosen fairly?
- Have any groups been excluded that could benefit from the research, because of non-scientific reasons, such as non-English or non-French speakers?
- Is the study population vulnerable in any way?

#### Favourable risk–benefit ratio

- Have the risks and benefits been assessed as accurately as possible?
- Is the risk–benefit ratio favourable?
- Are risks minimised?
- Are benefits maximised?

#### Independent review

- Where will the research be submitted for independent ethical review? Each university usually has an ethics committee responsible for this.
- Permission needed? Often the department of health or health facility has a process for giving permission to conduct the research.

#### Informed consent

- Has sufficient information been disclosed in a culturally- and linguistically-sensitive manner? The informed consent form should be written in simple, non-technical language and addressed directly to the person concerned.
- Waivers of informed consent? In some cases, such as the use of data obtained retrospectively from medical records for audit purposes, it may be impossible and unreasonable to find each patient in order to obtain informed consent. In situations such as this, a waiver of informed consent can be requested from the ethics committee.
- Children? Teenagers? Most countries have a legally-defined age at which children can consent to research. Below this age, informed consent must be obtained from the child's parent or legal guardian. Assent must still be obtained from the child. When your study population includes children, this can impose additional burdens on the research process in order to obtain consent.

#### Respect for participants and study communities

- Do the participants know they can withdraw from the study at any time?
- Have issues of confidentiality and privacy been addressed adequately? Participant names and personal details, such as telephone numbers, should not be sent to the statistician in the spreadsheet for analysis. Transcripts and quotations should not identify individuals. It may be necessary to protect confidentiality in data collection tools by the use of study codes rather than personal identifiers. Audiotapes of interviews should be stored securely and destroyed at a time specified in the proposal.

### Timetable

Write a simple timetable of how long it will take to complete each part of the research from start to finish. A Gantt bar chart is another useful way of presenting the timetable.^11^ This will include planning and gaining approval, data collection, data analysis and writing up. Be aware that planning and writing up often take longer than you anticipate. Think about how you will set aside time on a regular basis so as to complete the research project. The whole process can take two to three years, even for a relatively simple research project, thus students should plan their time accordingly. For example, you may need to schedule leave for data collection or writing up of your final thesis. Failure to complete the research assignment on time is a frequent reason for delay in graduating from the MMed programme.

### Funding and budget

Present a budget of the financial support that you need in order to complete the research and describe the potential sources of funding. Consider whether you should submit formal funding proposals to bodies such as the Medical Research Council. Most Masters level research does not require large-scale funding and funds can be provided by the researcher themselves or the university.

### References

List references to citations given in your proposal in an approved style, such as the Vancouver style, which is used in many journals.^12^ It is important to be very careful about following the exact formatting and content requirements for the style and for each type of reference you use, such as a book, journal article or internet source. In Vancouver style, the citation is a number in square brackets or in superscript that is inserted after the full stop at the end of the sentence. References are listed in numerical sequence, in the order in which they are first used in the text. It is recommended, if you have access, that you use a reference manager software, which can automatically create citations and reference lists in different styles. Look carefully at the reference list at the end of this article for examples of how to format a journal article, book chapter or internet source.

## Conclusion

This article introduces the series on primary care research and argues for the importance of building research capacity in the African context. The article presents an overview of the different types of primary care research and then focuses on the practical steps of choosing a research topic and writing the proposal. Future articles in the series will focus on different types of study design, data collection methods and data analysis. The final article will outline the writing of the thesis, research assignment or journal article.
